# A case report of bicornis bicollis uterus with unilateral cervical atresia: an unusual aetiology of chronic debilitating pelvic pain in a Cameroonian teenager

**DOI:** 10.1186/s12905-017-0396-9

**Published:** 2017-06-02

**Authors:** Julius Sama Dohbit, Esther Meka, Joel Noutakdie Tochie, Igor Kamla, Darolles Mwadjie, Pascal Foumane

**Affiliations:** 1 Faculty of Medicine and Biomedical Sciences, University of Yaoundé I, Yaoundé, Cameroon; 2 Department of Obstetrics and Gynaecology, Gynaeco-Obstetric and Paediatric Hospital, Yaoundé, Cameroon; 3 Department of Obstetrics and Gynaecology, Sangmelima Reference Hospital, Sangmelima, Cameroon

**Keywords:** Bicornuate uterus, Bicornis bicollis uterus, Cervical atresia, Chronic pelvic pain, Cameroon

## Abstract

**Background:**

Congenital uterine anomalies like bicornis or bicornuate uterus are relatively rare in sub-Saharan Africa. They are associated with an increased rate of spontaneous abortion, preterm delivery, and infertility. The occurrence of bicornis bicollis uterus with unilateral cervical atresia is exceptional and its management is controversial. We hereby report a rare cause of chronic pelvic pain in a Cameroonian teenager due to unilateral obstructive hematometra and hematosalpinx in the non-communicating horn of a bicornis bicollis uterus.

**Case presentation:**

A 13-year-old premenarchal non-virgin female presented with chronic and severe cyclical crampy pelvic pain. On clinical examination, she had a perforated hymen, a single vagina, and one uterine cervix. A two-dimensional pelvic ultrasonography revealed hematometra but missed out the underlying anomaly. Failure to drain the hematometra by serial cervical dilatations prompted an exploratory laparotomy which revealed: bicornis bicollis uterus with a right rudimentary uterine horn communicating with the vagina and a left non-communicating uterine horn distended by hematometra due to a homolateral cervical atresia. She underwent utero-vaginal canalization and a left hemi-hysterotomy with drainage of the hematometra. The postoperative period was uneventful. Regular cyclic menses occurred thereafter beginning at the first postoperative month. She had complete resolution of symptoms without recurrence after six months.

**Conclusion:**

Due to the risk of compromised fertility from bicornis uterus and the diagnostic challenges akin to resource-limited settings, we highlight the need for a high index of suspicion by healthcare providers when faced with chronic pelvic pain in premenarchal adolescents.

## Background

Bicornuate uterus (BU) is a congenital uterine anomaly with a prevalence of 0.4% in the general population [1]. It is often asymptomatic before puberty and thereafter has a significant association with infertility and miscarriage [1]. A bicornuate uterus consists of two symmetric uterine horns unified by caudal fusion. Both endometrial cavities communicate with the vagina either through a single uterine cervix (unicollis, most frequent) or through two uterine cervices (bicollis, less frequent). The occurrence of cervical atresia with BU is rare [2, 3]. Patients may present with pelvic pain due to hematometra and retrograde menstruation from a non-communicating uterine horn [2]. Treatment options are controversial and include hysterectomy or utero-vaginal canalization with the principal goal of relieving the symptoms and preserving fertility [3, 4].

Contrary to high-income countries where epidemiological studies have been carried out on these anomalies [1], the relative rarity of BU in sub-Saharan Africa (SSA) is due to a low index of clinical suspicion; under-reporting and inaccessibility to highly sensitive diagnostic tests like magnetic resonance imaging (MRI) [5, 6]. We herein, report an unusual case of bicornis bicollis uterus with unilateral cervical atresia in a Cameroonian teenager, presenting as chronic debilitating cyclical pelvic pain due to obstructive hematometra and managed by utero-vaginal canalization.

## Case presentation

A 13-year-old female student G_0_P_0_ presented with severe cyclical pelvic pain of gradual onset for a duration of 15 months. The pain was constricting in nature, predominant in the left iliac fossa, radiating to the back and perianal region and unremitting to analgesics and anti-spasmolytics. There was no fever, no urinary or gastrointestinal symptom. She had interrupted her education eight months prior to consultation due to the incapacitating nature of the pain.

She was premenarchal, had pubarche and thelarche at the ages of 9 and 11 years respectively. She had no chronic illness and had never undergone surgery. Her psychosocial and family histories were unremarkable. She had first coitus at the age of 11 years.

On examination, she was ill looking and had Tanner stage IV secondary sexual characteristics. Her temperature was 38.1 °C (100.58 °F) and other vital signs were within normal limits. Her conjunctivae were not pale. Abdominal examination revealed tenderness at the supra-pubic region and left iliac fossa without guarding. A moderately tender, rubbery, roughly spherical, supra-pubic pelvic mass of about 7 cm (longest diameter) was palpable. The physical appearance of her external genitalia was normal and she had a perforated hymen. On speculum examination, the vagina and cervix were normal. There was mild cervical motion tenderness, the pouch of Douglas was empty and the uterus was of normal size. Supra-pubic tenderness hindered clinical evaluation of the adnexae. Digital rectal examination and other systemic examinations were non-contributory. A working diagnosis of cervical stenosis complicated by cryptomenorrhoea was made and as differential diagnoses: chronic pelvic inflammatory disease and a pathologic ovarian cyst were advocated.

**Fig. 1:**
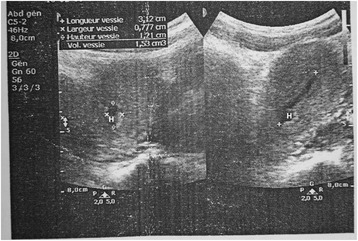
Showing Hydrometra (H) on two-dimensional pelvic ultrasound scan

**Fig. 2:**
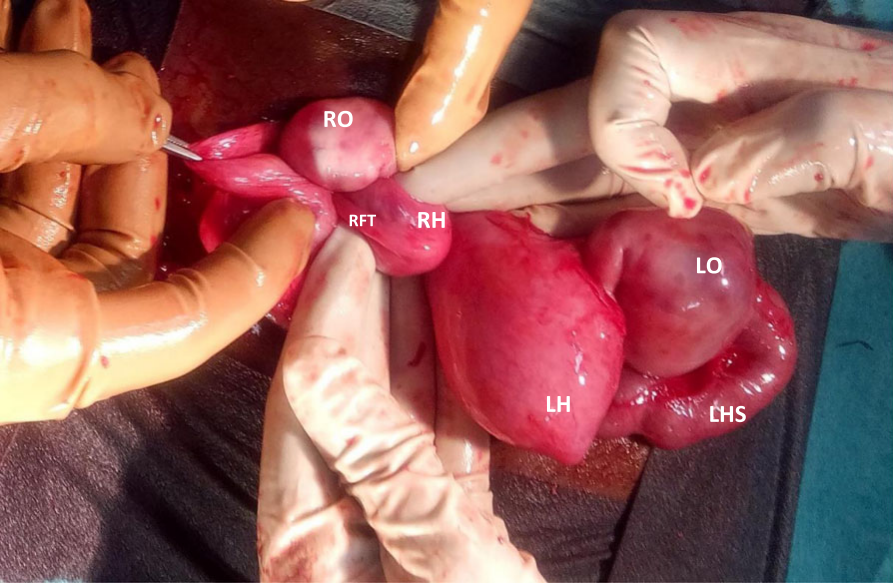
Bicornuate uterus with a left distented uterine horn (LH), right rudimentary uterine horn (RH), left hematosalpinx (LHS), right fallopian tube (RFT), congested left ovary (LO) and right ovary (RO)

**Fig. 3:**
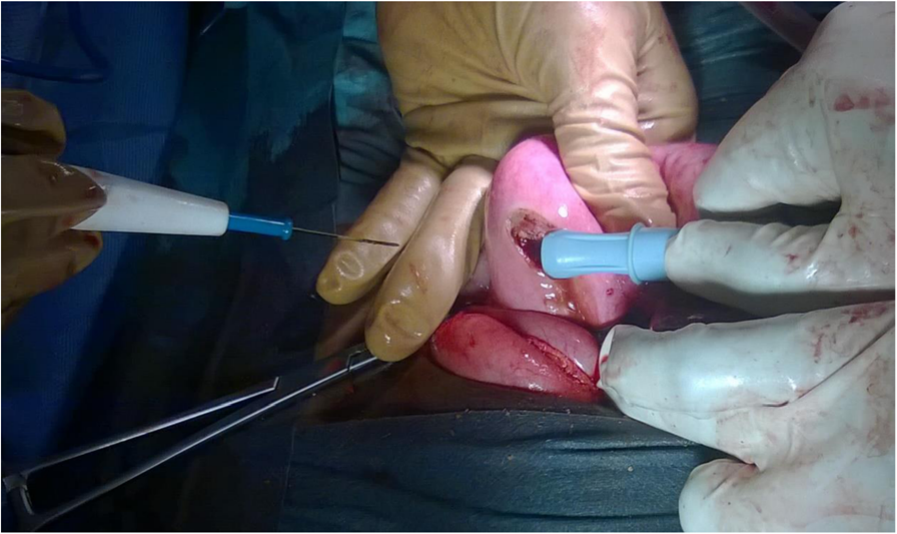
Left vertical corporeal hemi-hysterotomy and suction of ;the hematometra

A two-dimensional pelvic ultrasound scan demonstrated the presence of hydrometra of 225 ml (Fig. 1) and bilateral hydrosalpinx more predominant on the left side. She was admitted for an ultrasound guided cervical dilation under general anaesthesia. This procedure failed to drain any intra-uterine fluid despite easy intrauterine access with Hegar dilators. An intra-intervention ultrasound scan revealed the presence of a uterus of normal size and two lateral uterine masses. In the absence of an MRI scan in our setting to further explore the pelvis and these masses, the patient and her guardians were counselled and they consented to an exploratory laparotomy. Intra-operative findings were; a bicornis bicollis uterus with a left non-communicating uterine horn distended by hematometra due to left sided cervical atresia; a right atrophic rudimentary uterine horn communicating with the unique vagina (Fig. 2). Each horn had one ovary, one fallopian tube, one round ligament and one utero-sacral ligament. Both horns were separated at their base by a septum. The left ovary was hypertrophic and congested, while the right ovary was grossly normal. The left fallopian tube was tortuous and distended by blood, suggestive of hematosalpinx. Many tubo-uterine and tubo-ovarian adhesions were present. No other abdominal malformation was observed. Intra-operative management consisted of a left vertical corporeal hemi-hysterotomy of about 2 cm, which ensured drainage of approximately 300 ml of a tarry brown intra-uterine fluid (Fig. 3). The left uterine cavity was probed with Hegar’s dilators until felt through a fibrosed transverse membrane over the blind pouch into the vagina. The membrane was incised posteriorly, creating an ostium between the vagina and the left uterine cavity. A foley catheter was placed in the uterovaginal canal as a “stent”. The hysterotomy incision was closed in two layers. Both ovaries were conserved. The peritoneal cavity was washed with warm saline and the anterior abdominal wall closed layer by layer. The postoperative period was uneventful. The uterine catheter was removed on the fifth postoperative day and the patient was discharged on the sixth day with plans of follow-up cervical dilatation procedures. A postoperative abdominal ultrasound scan ruled out renal anomalies. She started regular monthly menstruation from the first postoperative month without dysmenorrhoea. She had complete resolution of symptoms without recurrence after six months.

## Discussion

Congenital uterine anomalies are incidental findings. A recent systematic review showed a prevalence of 5.5% in the general population, with a prevalence rate of 0.2%- 0.6% accounted by BU [1]. BU arises from an incomplete lateral fusion of the two Mullerian ducts at about the tenth week of intrauterine life from idiopathic causes [1]**.** The association of BU with cervical atresia is unusual because BU is often a non-obstructive type of Mullerian anomaly. Only sporadic cases similar to this one have been reported along with their surgical management [2, 3]. The occurrence of primary amenorrhoea associated with normal secondary sexual characteristics and cyclical pelvic pain (that worsens during puberty) should result in consideration of an obstructive congenital anomaly of this sort [7, 8]. Pelvic examination helps to rule out an imperforated hymen or a blind vaginal pouch, and may reveal a pelvic mass corresponding to hematometra, or hematosalpinx resulting from obstructed menstrual flow [8, 9]. Alternatively, patients may be asymptomatic in case the uterine horns are lined with non-functional endometrium [10] or symptoms may be non-specific, leading to late diagnoses and a high incidence of complications [11]. The resultant cyclical pelvic pain may be non-responsive to analgesics and severe enough to interfere with the patient’s quality of life, as seen in the above case [12, 13]. Our low index of clinical suspicion owing to the scarcity of data on the epidemiology and clinical presentation of this anomaly in SSA, coupled with sub-optimal diagnostic testing (two-dimensional ultrasonography) illustrate the frequent diagnostic challenges of congenital uterine anomalies encountered in resource-limited settings [5]. Three-dimensional ultrasound is increasingly being preferred over two-dimensional ultrasound for diagnosis because it provides a coronal view that enables an accurate description of the cervical anatomy as well as the differentiation and morphological classification of the various subtypes of Mullerian duct anomalies [14]. However, the high power of resolution of MRI makes it the imaging technique of choice for the diagnosis of uterine anomalies, its associated urinary tract malformations and in choosing the most appropriate surgical technique [8, 15]. Our patient could not benefit from an MRI scan because this sophisticated and expensive diagnostic test was inaccessible to her at the time of her management. At present, Cameroon has only three functional MRI machines for about 22 million inhabitants.

The treatment of BU with cervical atresia is controversial, consisting of surgical techniques like hysterectomy or utero-vaginal canalization [3, 4]. The latter is conservative surgery that entails the creation of a new endocervical canal through the fibrous tissues separating the uterus from the vagina, followed by putting in place of a stent often with a surrounding full or split-thickness skin graft to sustain epithelial ingrowth [4]**.** Historically, due to the high postoperative mortality rate and complications of utero-vaginal canalization, namely re-stenosis of the neo-ostium, low postoperative fertility rate and severe infection by ascending route, some authors advocate for hysterectomy even in young patients [2, 16]. However. utero-vaginal canalization is now being frequently performed due to recently reported uneventful postoperative outcomes, successful term deliveries after surgery and reduced psychological sequelae [4, 8, 17]. Some authors recommend that this surgery should be performed before puberty to avoid the risk of endometriosis from retrograde menstruation [8, 18]. Together with the patient’s family, we chose to be conservative and we performed utero-vaginal canalization considering the strong future pregnancy desires. Generally, the main aims of treatment are to relieve symptoms, to provide a conduit for regular menstruation and to preserve reproductive potential [7]. In our case, the short-term goal of treatment was attained; resolution of pelvic pain, initiation of regular menstruation (ruling out re-stenosis of the new cervical canal) and the absence of post-operative infection when she was last re-evaluated at the sixth postoperative month. Regular follow-up visits are however, recommended for early detection of any late postoperative complication.

Finally, fertility prognosis is debatable as successful term pregnancies by natural or artificial insemination have been reported after surgical correction [8, 10, 19]. However, fertility may be compromised by several other factors including the lack of cervical mucus, upper reproductive tract malformations, restenosis of the new cervix, postoperative adhesions and endometriosis caused by retrograde menstruation prior to surgery [7, 10, 20, 21]. Grimbizis et al. observed an overall 55.2% live birth rate, 40.6% term delivery rate, 36% spontaneous abortion rate, and 23% preterm birth rate in 261 women with BU [22]. Although there is currently no randomised controlled trial in favour or against surgical resection of the non-communicating horn of a Mullerian duct anomaly prior to conception in women with poor reproductive performances, several publications in the form of case reports and case series have shown successful postoperative term pregnancies following surgical resection of this uterine horn, preferably performed via laparoscopy [23–25]. Furthermore, resection of the non-communicating horn is often indicated if it is lined with functional endometrium, in order to avert catastrophic complications of horn pregnancies, ectopic pregnancies and endometriosis [26]. Hence in our case, resection of the right rudimentary horn was not absolutely indicated because of it was thought to be line with non-functional endometrium, given its absence of menstrual bleeding despite communication with the vagina. On the other hand, surgical resection of the left non-communicating uterine horn which presented with obstructive symptoms was indicated. However, because this horn carried the sole apparent probability of hosting a pregnancy in the present nulliparous patient, its resection was not performed due to refusal by her family. The patient and her family were counselled on the importance of regular clinical follow-up visits and to seek appropriate qualified obstetric evaluation in due course when pregnancy is desired.

## Conclusion

We have reported the first case of concomitant bicornis bicollis uterus with unilateral cervical atresia in Cameroon. Although limited resources precluded proper investigation with magnetic resonance imaging, we have shown that utero-vaginal canalization could yield favourable postoperative outcomes. This would need to be further explored in large multi-centre clinical trials in our setting. Health care personnel should have a high index of clinical suspicion for this congenital uterine anomaly as a potential differential diagnosis in premenarchal Cameroonian adolescents with unremitting cyclical chronic pelvic pain. Robust and advanced imaging modalities when available should help confirm clinical suspicion. The benefits of a timely diagnosis and treatment of such congenital uterine anomalies cannot be overemphasized in a setting already burdened with elevated female infertility.

## Abbreviations

BU: Bicornuate Uterus
MRI: Magnetic resonance imaging
SSA: Sub-Saharan Africa
